# Effectiveness of percutaneous lumbar foraminoplasty in patients with lumbar foraminal spinal stenosis accompanying redundant nerve root syndrome: A retrospective observational study

**DOI:** 10.1097/MD.0000000000021690

**Published:** 2020-08-14

**Authors:** Ki-Soon Jeong, Sung-Ae Cho, Woo-Suk Chung, Chi-Bum In

**Affiliations:** a Department of Anesthesiology and Pain Medicine; b Department of Radiology, Konyang University Hospital, Daejeon, Republic of Korea.

**Keywords:** lumbar foraminal spinal stenosis, percutaneous lumbar foraminoplasty, redundant nerve root syndrome

## Abstract

The clinical outcomes of redundant nerve root syndrome (RNRS) in patients with lumbar foraminal spinal stenosis (LFSS) are currently unknown. The purpose of this study was to evaluate the postprocedural outcomes of RNRS in LFSS after percutaneous lumbar foraminoplasty (PLF) and identify the factors associated with RNRS by comparative analysis between patients with and without RNRS.

Patients with LFSS who underwent PLF were retrospectively analyzed. RNRS is defined as the presence of thick, elongated, and tortuous structures in the cauda equine associated with lumbar spinal stenosis. Based on the sagittal or transverse magnetic resonance imaging scans obtained before the PLF, the patients were stratified into 2 groups. Comparative analysis was performed between patients with RNRS (group R) and those without RNRS (group C).

From March 2016 to January 2019, 8 of the 21 (38.1%) patients undergoing PLF showed signs of RNRS on magnetic resonance imaging images. PLF showed a tendency for less therapeutic effect with respect to changes in pain intensity in group R as compared to group C, but there were no statistically significant differences between the 2 groups. RNRS correlated with the cross-sectional area (CSA) of the dural sac and LFSS grade (*P* < .05). The CSA of the dural sac was smaller and the grade of LFSS was higher in group R than in group C.

RNRS is commonly associated with lumbar spinal stenosis and could affect the treatment outcomes. Clinical outcomes in group R were not statistically different from those in group C, although group R showed slightly worse outcomes. The independent factors associated with RNRS were CSA of the dural sac and the LFSS grade.

## 1 Introduction

Lumbar spinal stenosis (LSS) is defined as narrowing of the lumbar spinal canal.^[1]^ The word “stenosis” indicates narrowing of tubular structure in the body. LSS is mainly caused by disc herniation, facet hypertrophy, and ligamentum flavum buckling.^[1–3]^ These anatomical conditions lead to narrowing of the spinal and nerve root canal. Further, this anatomic stenosis can cause compression pressure on the lumbar spinal nerve root.

Redundant nerve root syndrome (RNRS) is defined as the presence of elongated, engorged, tortuous nerve roots in the lumbar subarachnoid space.^[2–5]^ The occurrence of RNRS is known to be closely related to LSS.^[5]^ Continuous compressive forces on nerve roots within the spinal canal can cause RNRS to occur.^[3]^ Although the etiology of RNRS is not yet clear, it is hypothesized that restriction of normal movement of spinal nerve roots in the constriction area leads to their stretching during spinal flexion and extension movement, thus causing their subsequent redundancy and elongation.^[2]^ As a result, a degenerative change of nerve roots can be induced.^[4]^

Percutaneous lumbar foraminoplasty (PLF) is one of the interventional options for the treatment of lumbar foraminal spinal stenosis (LFSS).^[6]^ LFSS is a specific type of LSS, defined as the narrowing or tightening of the nerve root exit in the intervertebral foramen.^[7–9]^ PLF is suggested as a minimally invasive procedures for treating of LFSS which has been refractory to conservative treatment such as oral medications, physical therapy, and epidural steroid injections.^[6,10]^ It has been reported that PLF using Claudicare, a specially designed instrument, is effective for pain reduction and functional improvement without complications.^[6]^

It is unclear whether RNRS is a simple phenomenon due to LSS or a pathological cause of symptoms such as lumbar radiculopathy.^[10]^ In addition, the clinical significance of RNRS in patients with LSS has been scarcely investigated. Lee et al^[5]^ demonstrated that lumbar epidural steroid injection was less effective in LSS patients with than without RNRS. The degenerative changes of nerve roots may reduce the effect of treatment. This observational, retrospective study was performed to evaluate the effects of PLF using Claudicare in patients with RNRS and identify the factors associated with RNRS.

## 2 Materials and methods

### 2.1 Patients

The Institutional Review Board of the Konyang University Hospital, Daejeon, Korea approved this study in December 2019 (No. KYUH 2019-12-004). The clinical data of patients (aged 20–90 years) with LFSS treated by PLF, from March 2016 to January 2019, were retrospectively analyzed. The inclusion criteria were

1. lumbosacral radiculopathy or referred leg pain,
2. LSS in one or more regions (subarticular, foraminal, or extraforaminal) confirmed by magnetic resonance imaging (MRI),
3. no more pain relief for other treatments (conservative treatment, oral medications, epidural steroid injections),
4. PLF at one or more levels (L3/4 to L5/S1).

The exclusion criteria were

1. history of PLF,
2. history of spinal surgery,
3. large disc herniation or severe central spinal stenosis on MRI,
4. segmental instability at the level of PLF,
5. spinal arteriovenous malformation on MRI, and
6. history of spinal cancer, fracture, or infection.

Their medical records were reviewed for patient demographics, duration of pain, intensity of pain based on the numeric rating scale (NRS), and spinal level at which PLF was performed. The level of spinal stenosis, LFSS grade, cross-sectional area (CSA) of the dural sac of central spinal canal, and distance from the conus medullaris to the most severe spinal stenosis level (distance from C to S) were recorded on the lumbar MRI.

### 2.2 Radiologic evaluation

**Figure 1:**
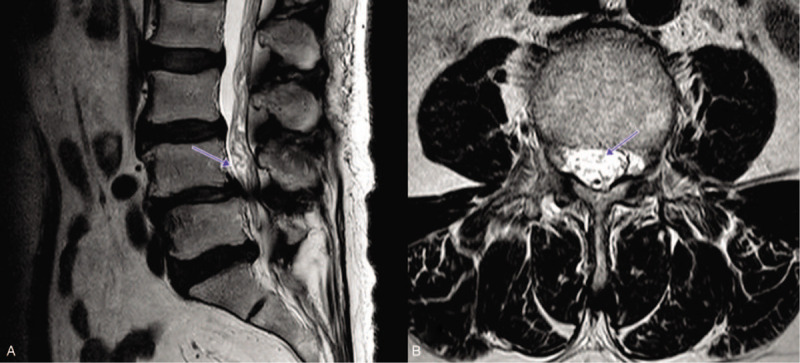
Elongated and tortuous nerve roots can be identified on sagittal (A) and axial (B) T2-weighted magnetic resonance imaging.

**Figure 2:**
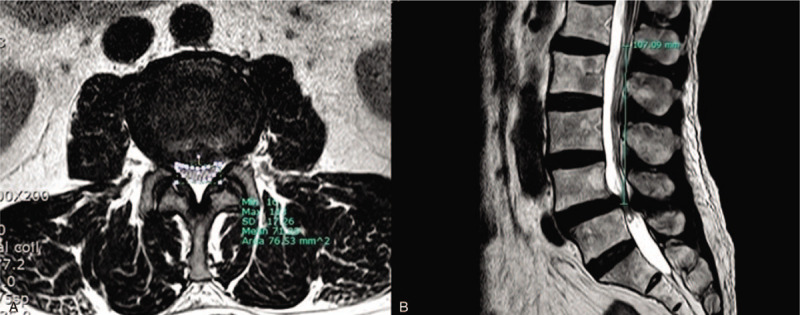
Illustration of the dural sac cross-sectional area and distance from conus medullaris to the most severe lumbar spinal stenosis level measurement technique.

The MRI imaging was analyzed by a pain clinic specialist and a radiologist with over 5 years of clinical experience. The presence of RNRS was reviewed on sagittal and axial T2-weighted MRI images by the radiologist. If there were elongated, engorged, tortuous nerve roots, RNRS was confirmed (Fig. 1). The level of spinal stenosis and grade of LFSS were recorded from the T2-weighted imaging. There were 4 grades of LFSS: grade 0 (normal), no abnormal finding; grade 1 (mild), perineural fat obliteration in 2 opposing directions (vertical or transverse) with no morphologic change; grade 2 (moderate), perineural fat obliteration surrounding the nerve root in 4 directions without morphologic change in both vertical and transverse directions; and grade 3 (severe), nerve root collapse or morphological change. The classification criteria for these grades were based on a paper on the MRI grading system for LFSS.^[11]^ At the most severe LSS level, measurement of the CSA of lumbar dural sac and the distance from C to S was performed using radiological workstation on MRI. The CSA of the dural sac was defined as the area distributed by the dural sac on axial T2 weighted images, and the distance from C to S was defined as the length from the conus medullaris to the most severe spinal stenosis level on mid-sagittal lumbar MRI scan (Fig. 2). The determination of grade of LFSS and the measurement of CSA and distance from C to S were performed by the pain clinic specialist who was blinded to the presence of RNRS. The MRI images to be measured were selected and provided by the radiologist.

### 2.3 Percutaneous lumbar foraminoplasty

**Figure 3:**
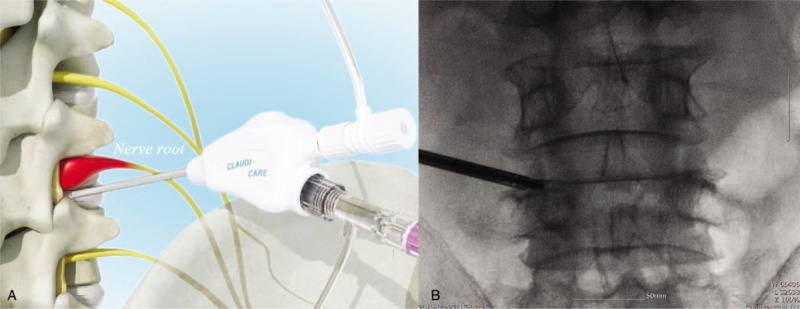
Schematic drawing of the Claudicare (A) and the final position of the working channel in the AP fluoroscope image (B).

The target level of PLF was determined based on patients’ symptoms and MRI imaging. Claudicare (Seawon Medi-Tech, Inc, Bucheon-si, Gyeonggi-do, Republic of Korea), which is a specially designed device ensuring improved safety during PLF, was used to perform PLF. This device consists of a guide wire (<1 mm in diameter), dilator (2 mm in diameter), working channel (inner/outer diameter: 3 mm/3.5 mm), and working drill.^[6]^ The drill was directed towards the superior articular process (SAP) in the intervertebral foramen under guidance of the wire, dilator, and working channel in order to use the fluoroscope. When the tip of the drill was located below the SAP, drilling and grinding of the hypertrophied SAP with thickened transforaminal ligament was performed (Fig. 3).

### 2.4 Clinical outcome evaluation and factors associated with RNRS

Patients were stratified into two groups: group R, patients with RNRS; and group C, patients without RNRS. The PLF was performed according to the patients’ symptoms and MRI findings. The primary outcome of this article was to evaluate the pain reduction using NRS in patients with RNRS after PLF. NRS pain score was assessed with 0 as the lowest score (no pain) and 10 as the highest score (the worst pain imaginable). It was assessed before and one week after PLF by a resident physician who did not know whether the patient had RNRS. The extent of the pain reduction was compared between the 2 groups based on the preprocedural NRS score. If the pain reduction was >30% or <30%, it was classified into “good response” group and “mild response” group, respectively. The secondary outcome was to evaluate the factors associated with pain reduction and RNRS based on medical records and lumbar MRI.

### 2.5 Statistical analysis

Continuous variables were presented as mean ± standard deviation or interquartile range (IQR). Categorical variables were expressed as frequency or percentage. The extent of pain reduction on the NRS (%) was compared between group R and group C using the student *t*-test. We assessed the proportion of good responses, which were defined as at least 30% decrease recorded on the NRS. The factors associated with good responses were then analyzed using the student *t*-test and Fisher exact test for parametric data. Furthermore, the independent factors associated with RNRS were analyzed using the student *t*-test and Fisher exact test. All statistical analyses were conducted using SPSS software (ver. 18.0 for Windows; SPSS Inc, Chicago, IL) and *P*-value <.05 was considered statistically significant.

## 3 Results

**Figure 4:**
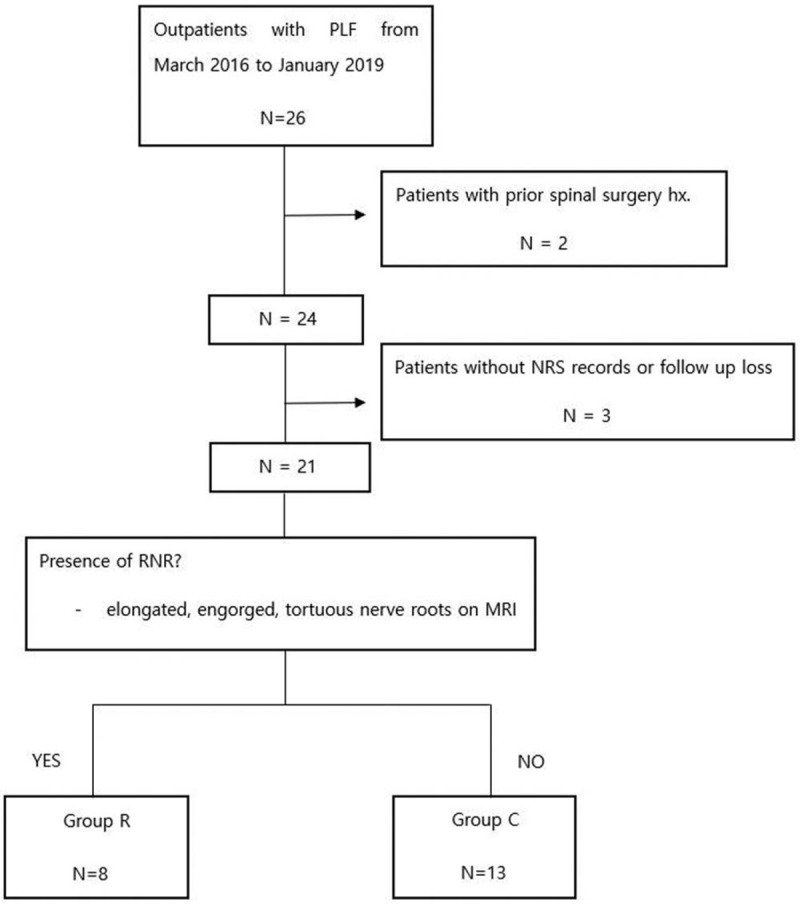
Study flow diagram.

We identified 26 patients who underwent PLF using Claudicare during the study period. Of the 26 patients, 5 were excluded due to a history of spinal surgery before PLF (n = 2) and absence of NRS records or loss of follow-up (n = 3). Finally, 21 patients were included in this study, of which 8 (38.1%) patients were identified to have RNRS on MRI. The final study group consisted of 21 patients, with 13 in group C and 8 in group R (Fig. 4).

There were no significant differences in patient demographics between the 2 groups in age, gender, intensity and duration of pain, levels of spinal stenosis except for levels of PLF. Spinal levels for PLF were mostly at L4/5 in group C and a high rate of simultaneous L3/4 and L4/5 in group R, with a statistically significant difference (*P* = .01; Table 1).

**Table 1:**
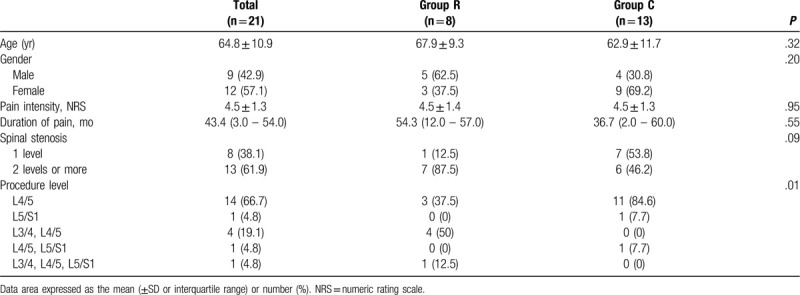
Patient characteristics.

**Figure 5:**
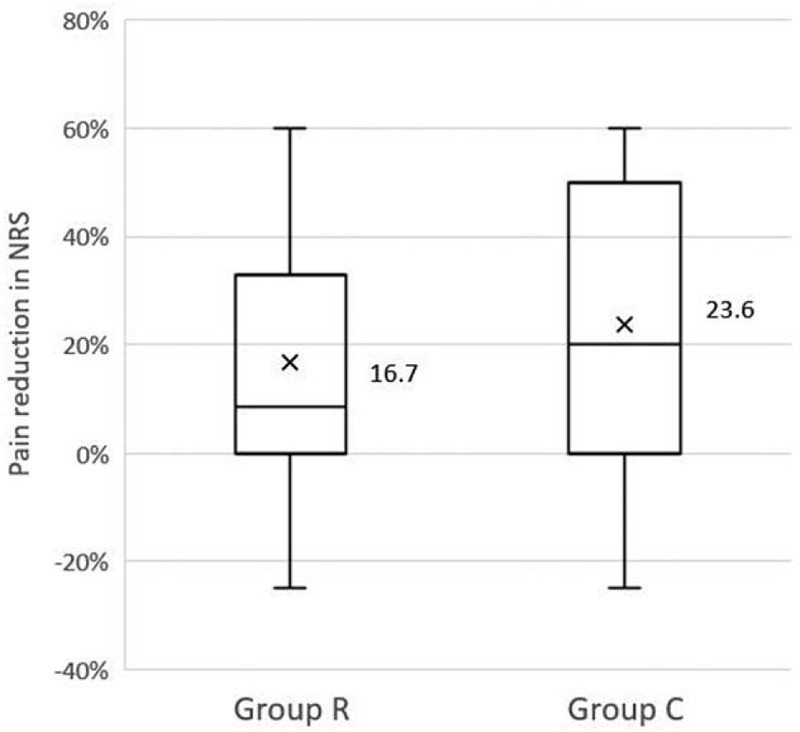
Percentage of pain reduction, a week after the procedure, based on preoperative pain score in patients with (group R) and without (group C) redundant nerve root syndrome. Graph shows the mean values (X) and interquartile range. The group R showed a less pain relief, but there was no statistical difference between the 2 groups.

PLF showed a tendency to have less effect with respect to the changes in pain intensity (△NRS, one week after PLF) in group R than that of group C, but there were no statistically significant differences between the 2 groups (16.7 [IQR 0-49.1] and 23.6 [IQR 0-53.6], respectively, *P* = .60; Fig. 5).

The proportion of RNRS among the “good response” patients after PLF was lower than that of “mild response patients” (28.6% and 42.9%, respectively; *P* = .66; Table 2). A comparison of the mild and good response groups after PLF showed no statistically significant difference in parameters of duration of pain, levels of spinal stenosis, CSA, and grade of LFSS. (Table 2)

**Table 2:**
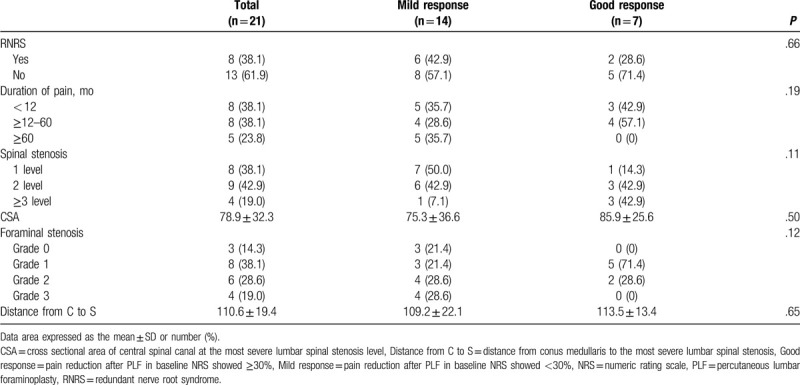
Factors associated with response of PLF.

**Figure 6:**
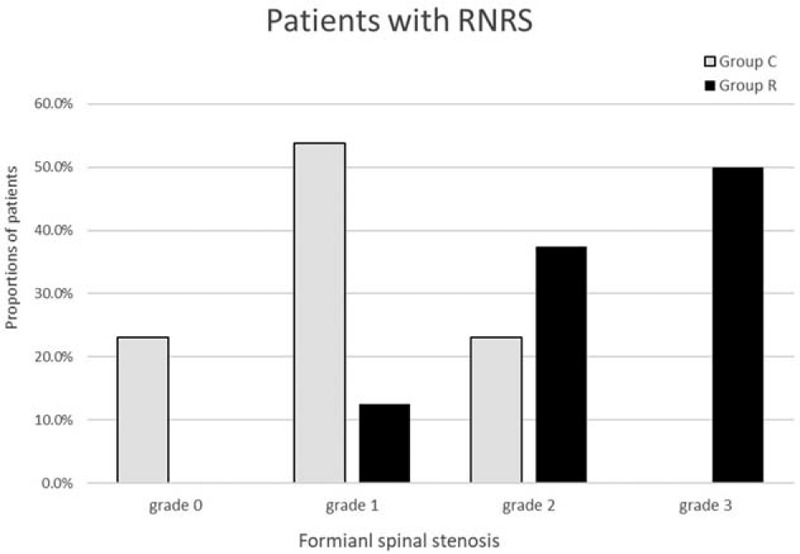
Proportions of patients according to grade of foraminal spinal stenosis in patients with (group R) and without (group C) redundant nerve root syndrome . As the grade of lumbar foraminal spinal stenosis increased, the proportion of patients increased in group R.

Table 3 demonstrates that RNRS was significantly associated with CSA and grade of LFSS. The CSA was smaller in group R than that of group C (59.7 ± 18.2 and 90.7 ± 35.1, respectively; *P* = .03). As the grade of LFSS increased, the proportion of patients increased in group R (*P* = .01; Table 3, Fig. 6). Duration of pain, levels of spinal stenosis, distance from C to S, and presence of claudication were not statistically associated with RNRS.

**Table 3:**
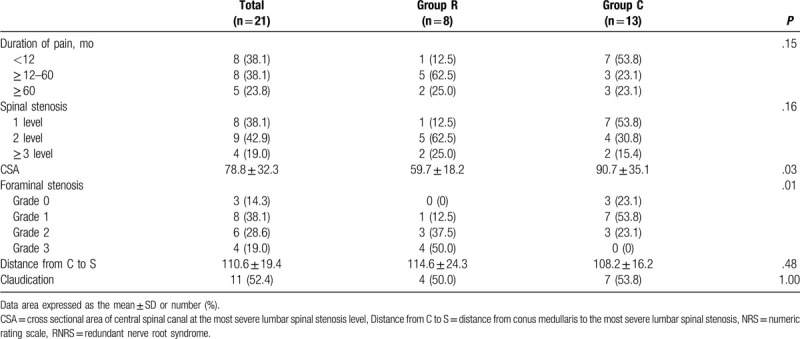
Factors associated with RNRS.

## 4 Discussion

The aim of the present study was to evaluate the clinical significance of PLF with Claudicare in LFSS with RNRS. We observed that the pain-reducing effect of PLF tended to be lower in patients with than without RNRS, but it was not statistically significant. In general, factors which may affect the outcome of PLF in patients with LSS include age, duration of pain, and level of spinal stenosis. In addition, Suzuki et al^[12]^ reported that the patients with RNRS were older in age, had longer symptom duration, and showed more severe signs and symptoms than patients without RNRS. Patients included in this study had relatively old age and long-lasting pain, but there were no statistically significant differences between the 2 groups. Also, The NRS pain scores before PLF and level of spinal stenosis did not show significant differences between the groups.

Earlier studies showed inconsistent results on the treatment outcome of the presence of RNRS in LSS. Some studies^[5,13]^ showed poorer outcomes associated with RNRS, while others^[14]^ reported no significant differences similar to our study. This result may have been influenced by several factors, including subject patients, assessment components, and treatment methods.

In this study, PLF was performed using Claudicare, which was specifically designed to treat LFSS. LFSS is defined as the narrowing of the nerve root exit,^[7–9]^ caused by a herniated lumbar intervertebral disc, osteoarthritic changes in the fact joints, and hypertrophied ligamentum flavum.^[15–17]^ The initial treatment of pain caused by LFSS can begin with conservative management. In the next step, various types of minimally invasive procedures are considered for patients with LFSS who do not respond to conservative treatment. Although percutaneous epidural adhesiolysis has been proposed as a minimally invasive procedure, a previous study reported a negative outcome for LFSS.^[18]^ In addition, percutaneous endoscopic lumbar foraminotomy has been suggested as a basic approach for treatment of LFSS.^[19,20]^ However, this endoscopic approach requires a steep learning curve for physicians and hospitalization of patients due to the occurrence of procedure-related adverse events. PLF was introduced in the mid-2000s to drill the tip of the SAP.^[19]^ Li et al^[10]^ reported that PLF is a less-invasive and effective surgery performed under fluoroscopic guidance without an endoscope and is convenient and time-saving.^[6,10]^ Our study used Claudicare, which has improved safety with the use of a tip with a blunted end and protective shield. This device can be used to drill the hypertrophied SAP to resect the foraminal ligament and partially enlarge the lumbar foramen in patients with LFSS. Yoo et al^[6]^ reported that PLF with Claudicare showed successful outcomes in 63% of patients, with decreased pain intensity and improved Oswestry disability index.

The occurrence of RNRS is thought to be closely related to LSS. Our study showed that patients with RNRS accounted for 38.1% of the total patients analyzed. The reported prevalence of RNRS ranges from 33.8% to 42% of patients with LSS,^[3]^ and its occurrence is considered relatively common in association with LSS. The etiology of RNRS can be attributed to the focal constriction of the nerve roots that restricts the normal movement of the spinal nerves, which causes stretching of nerve roots during flexion and extension of the spine. Thus, elongation and redundancy of the nerve roots occurs, accompanied by demyelination, endoneural fibrosis, and Schwann cell proliferation at the histological level.^[2,4,5]^ This degenerative change of nerve roots is not always reversible, and persistent RNRS is associated with poor outcomes.^[14]^ Yokohama et al^[13]^ reported that RNRS may resolve after operation, with better results observed for patients with than without RNRS resolution.

In our study, though less pain reduction in RNRS patients was observed, the clinical result of pain reduction in PLF is not significantly different in patients with than without RNRS. We expected RNRS to cause adverse results for the PLF, but there were no statistically significant differences. A few possible causes could explain the basis for this result. First, RNRS can be divided into 2 types.^[12]^ Type I of RNRS is a mild degree of tortuosity of nerve roots without thickening, whereas type II of RNRS is characterized by grossly thickened nerve roots, which is a rare and more severe form.^[5]^ Compared to type I, it is likely that type II is related to an irreversible state of nerve roots, therefore, the clinical outcome may vary depending on the ratio of type I to type II in the RNRS patients. In this study, the type of RNRS was not reflected in evaluating the results after PLF. Hence, further research on clinical results based on RNRS types is needed. Second, our study included a stenosis in subarticular area on the axial MRI among LFSS patients. LFSS can be classified into stenosis of extraforaminal, foraminal, and subarticular regions. Subarticular zone, also known as lateral recess and paracentral zone, is located in the most medial part of the axial image in LFSS. For anatomical reasons, lesions of subarticular zone are more difficult to perform PLF than those of foraminal and extraforaminal, and the outcome can be expected to be worse. In this study, patients with grade 0 of LFSS were subarticular type and were included only in the group without RNRS. As a result, the good response group did not include patients with grade 0 of LFSS, which may have contributed to reducing the effect of PLF in the patients without RNRS.

Although there were no statistically significant differences between the mild and good response groups in the present study, the following factors were likely to be associated with the good response group:

1. absence of RNRS,
2. short duration of pain (<60 months),
3. large CSA,
4. low grade of LFSS, and
5. longer distance from C to S.

The tendency of positive clinical outcome may have been affected by absence of RNRS and other factors which can reduce the occurrence of RNRS. Min el al^[14]^ indicated that there is a significant association between the distance from C to S (the length of redundant nerve roots) and the clinical outcome. The longer the distance from the C to S, the better is the outcome. It is likely that longer elongated nerve roots are able to withstand movements such as flexion and extension of the spine. This could have influenced the improvement of treatment outcomes.

Our study showed that RNRS was significantly associated with the CSA of dural sac and grade of LFSS. Meanwhile, the study by Lee et al^[5]^ reported that shorter the distance from C to S, more likely the occurrence of RNRS. This phenomenon can be explained by the shorter distance between these 2 points and lower capacity of the nerve root to adapt to the movement of the spine, thus, consequently higher chances of progression to RNRS. However, our study did not show this association. Instead, the CSA and grade of LFSS showed a statistically significant association with RNRS. The CSA was smaller and the grade of LFSS tended to be higher in patients with RNRS as compared to those without it. The pathogenesis of RNRS is most likely to be due to the squeezing force in the narrowed spinal canal applied on the nerve root.^[14]^ Thus, as the CSA of the spinal canal decreases, the force applied to the nerve root increases, resulting in a squeezing force. Meanwhile, other studies reported that the association between the CSA and RNRS was not significant, unlike in our study.^[5,13]^

The CSA of the spinal canal represents the degree of central LSS, and the grade of LFSS corresponds to the foraminal LSS. To our knowledge, no study has examined the association between the grade of LFSS and RNRS. In our study, the grade of LFSS tended to be significantly higher in the patients of RNRS. Accordingly, narrower the intervertebral foramen, chances of RNRS occurrence was higher. Considering the definition of pressure (force exerted per unit of surface area), if the pressures on the nerve root are similar, it can be assumed that spinal foramen with smaller CSA would have higher applied pressure per unit of nerve root than that of a spinal canal with larger CSA. This suggested that foraminal LSS could be more likely to influence the occurrence of RNRS rather than central LSS.

Moreover, the proximal third of the cauda equine could have a relative hypovascular region, called the watershed zone,^[1]^ which could lead to blood circulation problems.^[4]^ Compression-induced nerve root circulation impairment at the stenotic site is likely to cause RNRS. Although the existence of this hypovascular region is controversial, there is a possibility that the LFSS may have influenced this disturbance in circulation.^[1]^

A major limitation of this study is its retrospective nature. Small sample sizes and short follow-up periods are also drawbacks of this study. Although the results 1 week after PLF were compared to minimize the factors which may have affected the course of the treatment, such as the use of different drugs, the progression of pain reduction between the two groups may show different results as compared to this study over time. Another limitation is that there could be bias in selecting and measuring the MRI images. The presence of RNRS could not be completely hidden to the pain clinic specialist while measuring CSA and distance from C to S on MRI. Finally, there was no evaluation of various factors other than the pain reduction as part of clinical outcome in this study. An assessment of factors such as changes in the functional outcome after PLF, or improvement in claudication, one of the leading symptoms of LSS, is also required. Hence, further studies are needed in this direction.

## 5 Conclusion

Clinical outcomes associated with pain reduction were not statistically different between patients with and without RNRS, although patients with RNRS showed slightly worse outcomes. The independent factors associated with RNRS were the CSA of dural sac and grade of LFSS. The CSA was smaller and grade of LFSS tended to be higher in patients with RNRS than in those without RNRS. Further research is needed on the impact of RNRS on clinical outcomes and the associated factors affecting RNRS incidence.

## Acknowledgments

We acknowledge Jee-Young Hong and In-Seok Ko, Healthcare Data Science, Konyang University Hospital, Republic of Korea, as they supervised the statistical analysis of the work.

## Author contributions

Chi-Bum In: Conceptualization, Formal analysis, Methodology, Supervision, Writing - original draft, Writing - review & editing Ki-Soon Jeong: Conceptualization, Data curation, Investigation, Methodology, Writing - original draft, Writing - review & editing Sung-Ae Cho: Conceptualization, Data curation, Investigation, Methodology Woo-Suk Chung: Formal analysis, Investigation, Writing - review & editing.
